# Monitoring Copopulated Conformational States During Protein Folding Events Using Electrospray Ionization-Ion Mobility Spectrometry-Mass Spectrometry

**DOI:** 10.1016/j.jasms.2007.09.017

**Published:** 2007-12

**Authors:** David P. Smith, Kevin Giles, Robert H. Bateman, Sheena E. Radford, Alison E. Ashcroft

**Affiliations:** a Astbury Centre for Structural Molecular Biology, Faculty of Biological Sciences, University of Leeds, Leeds, United Kingdom; b Waters MS Technologies Centre, Micromass UK Ltd., Manchester, United Kingdom

## Abstract

The precise mechanism of protein folding remains elusive and there is a deficiency of biophysical techniques that are capable of monitoring the individual behavior of copopulated protein conformers during the folding process. Herein, an ion mobility spectrometry (IMS) device integrated with electrospray ionization mass spectrometry (ESI-MS) has been used to successfully separate and analyze protein conformers differing in cross section and/or charge state. In an initial test, an ensemble of folded and partially folded conformers of the protein cytochrome *c* was separated. A detailed study undertaken on the amyloidogenic protein β_2_-microglobulin (β_2_m), which forms fibrils by protein unfolding followed by self-aggregation and is responsible for the disease dialysis-related amyloidosis, has generated important insights into its folding landscape. Initially, a systematic titration of β_2_m over the pH range 2 to 7 using ESI-IMS-MS allowed individual conformers to be monitored and quantified throughout the acid denaturation process. Furthermore, a comparison of wild-type β_2_m with single and double amino acid variants with a range of folding stabilities and propensities for amyloid fibril formation has provided illuminating evidence of the role of different conformers in protein stability and amyloidogenic aggregation. The ESI-IMS-MS data presented here not only demonstrate an important and informative further dimension to ESI-MS, but also illustrate the potential of the ESI-IMS-MS technique for unravelling protein folding enigmas in general and studying protein misfolding diseases in particular.

The molecular details of how proteins fold efficiently to their unique three dimensional native conformations both in vitro and in vivo continues to present an intriguing challenge to biochemists and biomedical scientists despite extensive research [1, 2]. Integral to this problem are the difficulties in identifying and characterizing non-native states formed transiently during folding or unfolding, as such species are usually highly dynamic on the folding energy landscape [3]. In addition to providing new insights into the properties of native proteins and their non-native counterparts, identification of minor populated states is key to elucidating the molecular mechanisms involved in protein aggregation and consequent disease, and could lead to future remedies to counter amyloid disorders such as Alzheimer’s, Creutzfeldt-Jakob’s, and dialysis-related amyloidosis [2, 4, 5, 6].

Electrospray ionization mass spectrometry (ESI-MS) [7] has emerged as a powerful and important technique in the study of proteins; it not only enables the mass of a protein to be confirmed to within 0.01% error by the analysis of its (M + nH)^*n*+^ ions according to their *m/z* ratios, but also provides information regarding a protein’s conformational state from the number of charges detected on its ions and the width of its charge state distribution [8, 9, 10, 11, 12]. Indeed, copopulated protein conformers can be identified in some cases if each population gives rise to a unique charge state distribution, and for those proteins whose conformers give rise to overlapping charge state distributions, deconvolution methods have been developed and applied to estimate the relative proportions of each conformer present under different solution conditions [13, 14].

Conversely, ion mobility spectrometry (IMS) has the capability of separating ions of the same *m/z* ratio but with different collision cross sections and/or charge states by utilizing the mobility of an ion in a background gas under the influence of an electric field [15, 16, 17]. For example, in the case of copopulated protein conformers, a more folded conformer with a smaller cross section would have a higher mobility and hence a shorter drift time than more extended, less folded conformers of the same protein. Furthermore, two charge states arising from the same protein conformer may be separated, with the more highly charged species having a shorter drift time. Thus, a combination of ESI-MS and IMS (i.e., ESI-IMS-MS) has the extraordinary potential to permit the resolution and identification of components of a mixture that cannot be separated by MS alone, nor can be identified unambiguously by IMS. Consequently, ESI-IMS-MS offers the unique and exciting opportunity to analyze and quantify copopulated protein conformers and monitor their individual appearance and disappearance during the protein’s folding and unfolding processes [15, 16, 18].

A novel approach to the separation of gas-phase ions by mobility using a travelling voltage wave incorporated in a RF ion guide has been reported recently [19, 20]. This device, situated in between the analyzers of an ESI hybrid quadrupole-orthogonal acceleration time-of-flight (oa-TOF) mass spectrometer, has shown high transmission efficiency coupled with a separative power comparable with conventional IMS drift cell approaches in a number of applications, including the resolution of peptide monomer, dimer, trimer and tetramer ions of the same *m/z* ratio and the separation of isomeric peptides [19, 20]. In addition, the capability for collision cross section estimation has been illustrated for the noncovalently bound, macromolecular TRAP protein complex [21].

Here we have investigated the applicability of ESI-IMS-MS for the analysis of copopulated protein conformers to gain new insights into the structures, folding pathways and behavioral properties of two proteins: cytochrome *c*, a widely available protein that has been the subject of many ESI-MS [22, 23, 24, 25] and IMS-MS [26, 27] studies, and the amyloidogenic protein β_2_-microglobulin (β_2_m), which we have studied extensively by a plethora of biophysical techniques [28, 29, 30, 31, 32, 33] including mass spectrometry [14, 34, 35] and high field asymmetric waveform IMS (FAIMS) coupled to ESI-MS [36]. These preliminary ESI-IMS-MS analyses have yielded fascinating insights into protein folding pathways and intermediates and we have demonstrated the ability of this technique to resolve and quantify the population of coexisting conformational states of these proteins formed by acid denaturation. In particular, the cytochrome *c* studies have generated data compatible with previous findings using conventional IMS-MS [26] whilst copopulated β_2_m conformers have been resolved and non-native species, whose population may be linked to the enhanced amyloidogenic potential of these sequences, revealed at neutral pH for β_2_m variants.

## Methods

### Protein Preparation

Recombinant β_2_-microglobulin and variants were prepared as described previously [29, 33] and dialyzed into Milli-Q water (Millipore UK Ltd., Watford, UK); cytochrome *c* from equine heart was purchased and used as supplied from Sigma-Aldrich (Gillingham, Dorset, UK). The proteins were dissolved at a concentration of 8 pmol μL^−1^ in 50 mM aqueous ammonium acetate acidified with formic acid to pH 3, or in 1:1 vol/vol 10 mM ammonium formate:10 mM ammonium acetate acidified to the required pH with hydrochloric acid [14], as stated. All reagents were purchased from Sigma-Aldrich (Gillingham, Dorset, UK). Buffers were prepared in Milli-Q water (Millipore UK Ltd., Watford, UK).

### ESI-IMS-MS Analyses

Samples were infused into the ESI source of a Synapt HDMS, a hybrid quadrupole-IMS-orthogonal acceleration time-of-flight mass spectrometer (oa-TOF) [19, 20] (Waters UK Ltd, Manchester, UK) using a Harvard syringe pump (model 22; Harvard Apparatus, Holliston, MA) with a flow rate of 10 μL min^−1^. An ESI capillary voltage of 2.8 kV was used, together with a sampling cone voltage of 75 V. A source temperature of 80 °C and a desolvation temperature of 150 °C were set. Nitrogen was used as both the nebulizing gas and the desolvation gas. The quadrupole was operated in nonresolving mode to transmit a wide *m/z* range.

The ion mobility device in the Synapt HDMS consists of three travelling wave (T-wave) ion guides [19]: the first is used to store ions before mobility separation (trap T-Wave), the second provides ion mobility separation using a travelling voltage wave (IM T-Wave), and the third transfers the mobility-separated ions to the time-of-flight analyzer for *m/z* analysis (transfer T-Wave). In these experiments, the IM T-Wave was operated at a nitrogen pressure of 0.5 mbar and the trap/transfer T-Waves at a nominal pressure of 4 × 10^−2^ mbar (1:1 vol/vol nitrogen:argon). The ion accelerating voltages into the trap and transfer T-wave devices were set at 5 and 3 V, respectively. Each individual mobility experiment was 18 msec long with the 300 ms^−1^ IM T-Wave pulse amplitude being ramped from 7 to 13 V in this time; this ensured that the entire range of components was detected within a single experiment. During each mobility separation, ions were accumulated in the trap T-Wave and then released over a period of 90 μs into the IM T-Wave to start the next mobility separation. The transfer T-Wave had a 300 ms^−1^ 3 V pulse running continually to transfer the mobility separated ions to the oa-TOF whilst maintaining the temporal separation. Ion arrival time (mobility) spectra were recorded through synchronization of the gated release of ions for mobility separation and the oa-TOF mass spectral acquisitions. An individual mobility experiment was made up of 200 sequential oa-TOF mass spectra (90 μs each) giving an overall time of 18 ms (200 × 90 μs). Mass spectra were acquired over a *m/z* range of 500 to 3000 and with repeat 2s acquisition times per point (ca. 111 summed individual mobility experiments).

Instrument control and data analysis were performed using MassLynx ver. 4.1 software (Waters UK Ltd, Manchester, UK). Mobility data were visualized and processed using the Driftscope module within MassLynx. Mass accuracy was ensured by calibration on a separate introduction of sodium iodide (2 mg mL^−1^ in 1:1 vol/vol aqueous methanol).

### Deconvolution of ESI-IMS-MS Data

Drift time versus intensity (ion count) values for each charge state at its respective *m/z* value were extracted from the Driftscope plots available within the MassLynx suite of software and subsequently imported into Origin Pro 7.5 (Originlab, Northampton, MA). Plots were then fitted to a minimum number of Gaussian distributions using multiples of the following equation:

$$y=y_{o}+\frac{A}{w\sqrt{\pi /2}}\,\;e^{-2\frac{{(x-x_{o})}^{2}}{w^{2}}}$$

where *y_o_* = base line off-set, *A* = total area under the curve from the baseline, *x_o_* = center of the peak, and *w* = width of the peak at half height. This model describes a bell-shaped curve akin to the normal (Gaussian) probability distribution function. The center *x_o_* represents the “mean”, while *w*/2 is the standard deviation.

## Results and Discussion

### Cytochrome *c*

#### Detection and separation of cytochrome *c* conformers

Cytochrome *c* is an N-acetylated single domain protein of 104 amino acid residues with a covalently attached haem group [37]. This protein has been studied extensively by ESI-MS and IMS-MS over the past 17 years: thus it is an ideal protein to test the ability of ESI-IMS-MS to resolve multiple conformational states of the same polypeptide chain. In 1990, it was demonstrated that under different solution conditions, the ESI-MS spectra of cytochrome *c* contained different charge state distribution patterns representative of the different protein conformations existing in solution: viz., a narrow distribution of lowly charged species consistent with a folded protein accompanied by a wider distribution of more highly charged species arising from less folded conformational states [22]. After this, the refolding of acid-denatured cytochrome *c*, which illustrated the interconversion of these two charge state distributions, was monitored by on-line ESI-MS [23], and elsewhere methanol-induced partially folded states have also been detected [24]. Both conventional IMS [26, 38, 39, 40] and high field asymmetric waveform IMS (FAIMS) [41] coupled to ESI-MS have shown evidence for the existence and separation of multiple, copopulated conformers of cytochrome *c*. Indeed, five distinct, so-called “conformation families” have been described for cytochrome *c* in terms of their charge state distributions and cross section areas as a result of intensive ESI-IMS-MS studies with a conventional IMS device: two of these conformation families had measured cross sectional areas which correspond well to cross sectional areas calculated for the natively folded protein using two different methods, whilst the remaining three conformational types were consistent with more extended, less folded protein structures [26].

**Figure 1:**
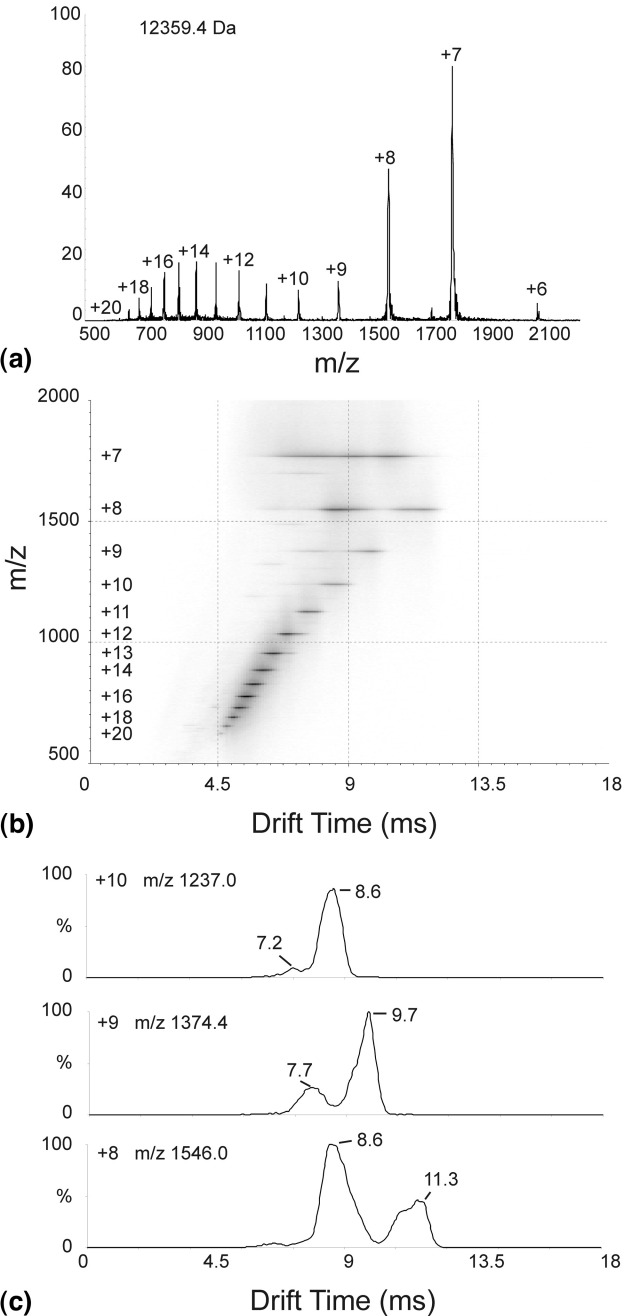
(**a**) The summed *m/z* spectrum from an ESI-IMS-MS data acquisition of cytochrome *c* analyzed in 50 mM aqueous ammonium acetate solution acidified to pH 3 showing a charge state distribution from +6 to +20 ions consistent with monomeric protein (12,359.4 Da). (**b**) ESI-IMS-MS Driftscope plot showing drift time (x axis) versus *m/z* (y axis) for the analysis of cytochrome *c* in 50 mM aqueous ammonium acetate solution acidified to pH 3. (**c**) ESI-IMS-MS drift time versus intensity graphs for the *m/z* 1237.0 (+10 ions; upper), *m/z* 1374.4 (+9 ions; middle), and *m/z* 1546.0 (+8 ions; lower) signals detected during the analysis of cytochrome *c* in 50 mM aqueous ammonium acetate solution acidified to pH 3.

As a preliminary assessment to detect and differentiate between copopulated protein conformers, ESI-IMS-MS analysis of cytochrome *c* in aqueous ammonium acetate buffer acidified to pH 3 was accomplished by infusion of the sample into the ESI source of the Synapt HDMS mass spectrometer (see the Methods section). Subsequent IMS separation of the charged species thus generated was followed by *m/z* analysis using the time-of-flight analyzer of the instrument [19, 20]. Summation of the total dataset acquired led to an ESI *m/z* spectrum in excellent agreement with spectral data produced under standard ESI-MS conditions, i.e., all ions detected under standard ESI-MS conditions were detected by ESI-IMS-MS. The total, combined spectrum of the dataset contained a charge state distribution consistent with monomeric cytochrome *c* encompassing ions ranging from the +6 (*m/z* 2061.1) to the +20 (*m/z* 619.0), Figure 1a. The presence of more than one charge state distribution implies a mixture of folded and less folded protein conformers, or conformational families, as expected [22], and the measured molecular mass 12,359.4 Da is in close agreement with the calculated mass (12,360.1 Da) for the N-acetylated protein with a haem group attached.

ESI-IMS-MS data processing involved the generation of three-dimensional *m/z* versus drift time versus intensity (Driftscope) plots (Figure 1b) and drift time versus intensity plots for selected *m/z* values (Figure 1c), from which the number of distinct species associated with each *m/z* value (and hence charge state) can be readily visualized. These figures do not take into account the fixed shift in drift times observed due to the transit time of a single wave through the IMS stage and the subsequent transfer optics to the oa-TOF analyzer. As this shift is in the order of ∼1 ms, it does not change the data or conclusions materially. On examination of the drift times of the +7 to +20 charge state ions relating to monomeric cytochrome *c* (Figure 1b), two major populations are apparent, one consisting primarily of +7 (drift time ∼ 9.9 ms), +8 (drift time ∼ 8.6 ms), and +9 (drift time ∼ 7.7 ms) charge state ions and the other of +8 (drift time ∼ 11.3 ms), +9 (drift time ∼ 9.7 ms), +10 (drift time ∼,8.6 ms), +11 (drift time ∼ 7.7 ms) etc. down to +20 (drift time ∼ 4.7 ms) charge state ions. The intensity versus drift time plots for the *m/z* 1546.0, 1374.4, and 1237.0 ions (corresponding to +8, +9, and +10 charge state ions, respectively) have been selected to illustrate these data further (Figure 1c). The +8 ions show two distinct signals with drift times of 8.6 and 11.3 ms, the +9 ions show two signals with drift times of 7.7 and 9.7 ms, and the +10 ions show a predominant signal with a drift time of 8.6 ms together with a signal of very low intensity with a drift time of 7.2 ms. In each case, the signal with the shorter drift time (i.e., higher mobility) can be ascribed to a more compact, more folded protein conformation compared with the signal detected at greater drift time (i.e., lower mobility) which can be assigned to a less folded, more extended conformation. Thus the +8 ion species exhibit a higher proportion of the folded conformation compared with the less folded species, whilst, in contrast, for the +9 and +10 ion species the less folded species predominates. These observations appear to be rational based on previous cytochrome *c* MS studies [22, 24, 26, 38, 39, 40, 42]. The +7 charge state ions also appear to contribute to other, minor species of shorter drift times (6.3 to 9 ms), which may well be consistent with the existence of multiple native-like conformations reported by others [26].

### β_2_-Microglobulin

#### Detection of copopulated β_2_-microglobulin protein conformers during acid unfolding

**Figure 2:**
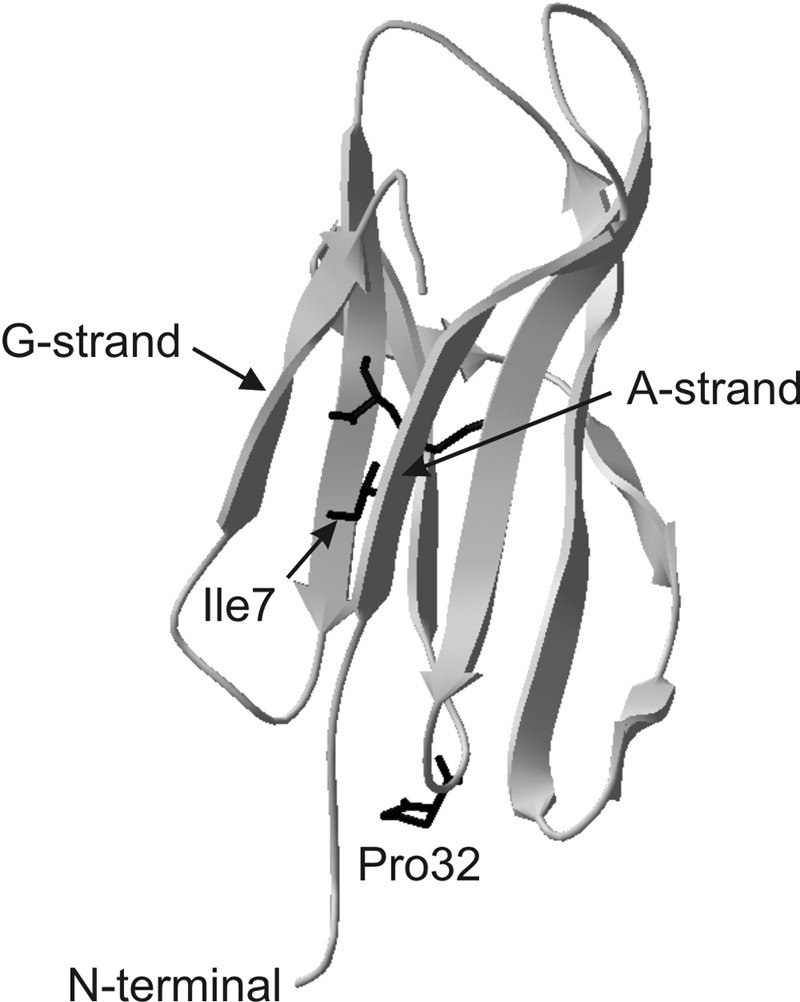
Topology diagram of the secondary structure of native β_2_-microglobulin (β_2_m). The location of the secondary structural elements was assigned from the crystal structure (PDB entry 1DUZ).

β_2_-Microglobulin (β_2_m) forms the light chain of the major histocompatibility Class I antigen [43]. The monomeric protein is found in serum at low concentrations [44] but in patients with renal failure the concentration of β_2_m can increase ∼50-fold, resulting in the formation and deposition of amyloid fibrils in the musculo-skeletal system [45]. The latter can lead to the human amyloid disease dialysis-related amyloidosis, which affects more than 700,000 patients world-wide [46]. Recombinant β_2_m is a 100-amino acid protein of 11,860.4 Da (including the N-terminal initiating methionine) with a seven-stranded β-sandwich structure and a single disulphide bridge (Figure 2). Recent ESI-MS studies have shown how the overall shape and extent of the charge state distribution generated by analysis of β_2_-microglobulin (β_2_m) varies according to the pH at which the protein is analyzed [14]. Deconvolution of these charge state distributions by Gaussian curve fitting indicated overlapping distributions relating to three different protein conformational types, the relative intensities of which depend on the pH of the sample analyzed. From these data the percentage populations of the folded, partially folded and acid-unfolded states of β_2_m over the pH range 6.0 to 2.0 were estimated and the results indicated that the native state is highly populated in the region pH 6.0 to 4.5, a partially folded state becomes populated at pH < 4.5 and an acid-unfolded state becomes populated at pH ≤3.5 [14]. The ESI-MS results were in good agreement with data obtained from a range of other techniques including CD [33], NMR [31, 32, 47] and crystallography [48]. NMR studies have shown that the partially folded state has a native-like conformation retaining significant stable structures in strands B to F, whilst the N-terminal A-strand of the protein is entirely unstructured and the C-terminal G-strand is highly destabilized (Figure 2) [28]. By contrast, the acid-unfolded conformation is known from NMR relaxation studies to be more highly denatured, containing no residual secondary structure, but with collapsed clusters still stabilized by the disulphide bond [47].

However, despite these promising ESI-MS results [14], the deconvolution of overlapping charge state distributions is difficult unless assumptions are made concerning the “permitted” width of the distribution and the optimum number of Gaussian distributions to be used for the fitting process. Also, whether or not more than one conformational species copopulates exactly the same charge state distribution as another, similar conformer cannot be determined directly by ESI-MS alone. Despite these limitations of ESI-MS, it is of interest to note that the number of species that comprise these conformational ensembles is virtually impossible to determine by other biophysical techniques including optical and spectroscopic methods.

Whilst native β_2_m is not able to form amyloid fibrils when incubated at neutral pH in the absence of seeds in vitro [31, 49], both the partially folded and the acid-unfolded states are amyloidogenic in vitro and self-assemble rapidly and spontaneously, forming amyloid-like fibrils with specific morphologies dependent on the precise experimental conditions employed [50]. The relative population of these amyloid-forming conformational states is a determining factor in the final fibril morphology [33] and the population of states with similar properties to the partially folded and acid-unfolded states is required for fibril formation under neutral pH conditions, as exemplified with a series of destabilized β_2_m mutants [28, 51]. Identifying the amyloid precursor from these heterogeneous ensembles represents a significant challenge.

**Figure 3:**
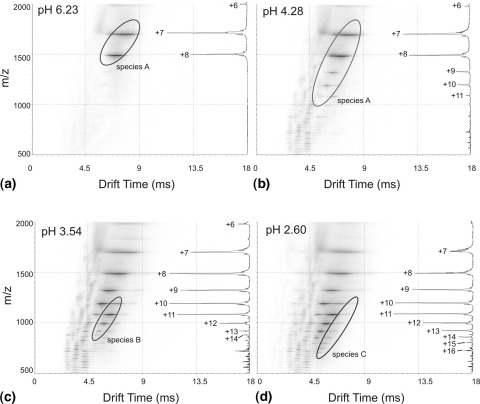
ESI-IMS-MS Driftscope plots showing drift time (x axis) versus *m/z* (y axis) for wild-type β_2_m analyzed at (**a**) pH 6.23, (**b**) pH 4.28, (**c**) pH 3.54, and (**d**) pH 2.60. The number of conformeric species observed for each individual charge state can be seen. Insets at the right hand side of each plot: the summed, full scan *m/z* spectra of wild-type β_2_m for each data acquisition showing the charge state ions detected.

To investigate the conformational heterogeneity of β_2_m under different conditions, ESI-IMS-MS analyses were undertaken in aqueous, buffered solution (10 mM ammonium acetate:10 mM ammonium formate) at regular intervals throughout the pH range 6.8 to 2.5. For each ESI-IMS-MS analysis, summation of the total data acquired led to *m/z* spectra in close agreement with spectral data previously produced under identical ESI-MS conditions but in the absence of IMS [14], and the mass errors were in all cases ≤0.01%. The summed *m/z* spectra are shown in Figure 3 (insets), together with Driftscope plots for the ESI-IMS-MS analysis of β_2_m at four key points during the pH titration: pH 6.23, 4.28, 3.54, and 2.60. At pH 6.23, β_2_m is fully folded [31, 48] and the summed *m/z* spectrum and the Driftscope plot are dominated by the +7 (*m/z* 1695.3) and +8 (*m/z* 1483.6) charge state ions, accompanied by a weak signal from the +6 (*m/z* 1977.7) charge state ions (Figure 3a). This is consistent with a folded conformer [14, 36] that will be referred to as Species A.

Upon acid titration with HCl to pH 4.28, the summed *m/z* spectrum shows an expanded charge state distribution which covers the +6 to +11 charge states consistent with the monomeric protein; the +7 and +8 charge state ions still dominate the spectrum, but additional signals of lower intensity have now been detected for the +9 (*m/z* 1318.8), +10 (*m/z* 1187.0), and +11 (*m/z* 1079.2) charge state ions (Figure 3b, inset). The corresponding Driftscope plot has increased in complexity: the signals for the +7 and +8 charge state ions remain unchanged at the same drift times, whilst signals for the +9, +10, and +11 charge state ions are now apparent. The +10 and +11 ions clearly indicate the presence of two conformers. For each of these charge states, the peaks with the shorter drift times are “in-line” with the +7 and +8 charge state ions associated with the folded conformer (Species A). The peaks with the longer drift times indicate the presence of a more expanded conformer (Species B; for clarity this has been labeled only in Figure 3c). The query arising from this Driftscope plot is whether the charge state distribution associated with the folded conformer in Figure 3a has increased from being comprised of the +7 and +8 charge state ions to now incorporating a series of +7 to +11 charge state ions due to either more extensive protonation of the protein or to a partial unfolding event leading to a little-changed, native-like conformation, or alternatively if the folded conformer is unchanged but is now accompanied by a second conformer that encompasses the +9, +10, and +11 charge state ions. If the latter is correct, then this second conformer must be of very similar cross sectional area to the folded conformer as the additional signals appear “in-line” with the +7 and +8 charge state ions of Species A (Figure 3b). The weak, regularly spaced signals of *m/z* <1000 relate to background ions and traces of low mass impurities arising from the solvents and buffer.

Acidification of β_2_m to pH 3.54 results in further protein unfolding, which is reflected in the expansion of the *m/z* charge state distribution that now includes contributions from the +6 to +14 (*m/z* 848.2) ions in the summed *m/z* spectrum (Figure 3c inset). At this pH the presence of the native-like conformer (Species A) is still apparent (in particular the +7 and +8 charge state ions). The signals associated with Species B are now more pronounced (+10 to +13 charge state ions) in the Driftscope plot (Figure 3c), and are accompanied by a weak signal consistent with a more extended conformer of lower mobility encompassing charge state ions +10 to +14 (Species C; labeled only in Figure 3d for clarity). Finally, acidification of β_2_m to pH 2.60 results in the further population of the acid-unfolded state [14, 31] and is reflected here with further expansion of the *m/z* charge state distribution (+7 to +17 ions). The Driftscope plot shows a diminishment of the more compact Species A, relative to the less folded Species B and C (Figure 3d).

**Figure 4:**
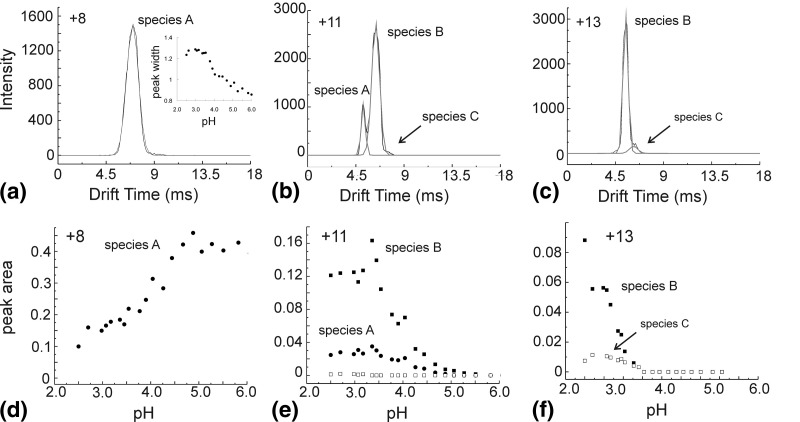
(**a**)–(**c**): Drift time versus intensity graphs for data acquired at pH 2.60 for (**a**) +8 charge state ions (*m/z* 1483.5), (**b**) +11 charge state ions (*m/z* 1079.2), and (**c**) +13 charge state ions (*m/z* 913.3). The Gaussian distribution fits are shown as grey curves. The inset in (**a**) shows the extent of broadening (in ms) of the +8 charge state ions observed with decreasing pH. (**d**)–(**f**): pH versus peak area graphs showing the protein conformeric species associated with (**d**) +8, (**e**) +11, and (**f**) +13 charge state ions. The peaks assigned to Species A are shown as ●, those assigned to Species B as ■, and those assigned to Species C as □. The peak areas were calculated from the Gaussian distributions (see the Method section) and are displayed as a fraction of the total peak area from the spectrum of origin.

To elucidate the ESI-IMS-MS data further and to compare these results with data obtained from other biophysical techniques on the same protein, we have estimated the proportion of each conformer contributing to the individual charge state ions at a given pH, by measurement of the drift time (mobility) peak areas for those charge state ions making a contribution to that conformer. In the case of partially resolved conformers, the drift time versus intensity (ion counts) plot at a given pH and *m/z* value was fitted to the minimum number of Gaussian distributions to enhance definition of the less well resolved components (see the Methods section). Both the mean value and the peak width were allowed to “float” during the fitting process. Examples of this fitting procedure for the +8, +11, and +13 charge state ions at pH 2.60 are shown in Figure 4a, b, and c, respectively. For the +8 ions (*m/z* 1483.6), the broad peak with a drift time of ∼6.9 ms was measured as a single entity (Figure 4a), although when comparing the drift time (mobility) signals originating from the +8 ions at each step of the pH titration, the peak width can be seen to broaden considerably as the pH of the solution decreases (Figure 4a, inset) (as is the case, but to a lesser extent, with other charge state ions; data not shown), thus suggesting that whilst at pH ∼ 6 these ions appear to be homogeneous, at lower pH they may well contribute to more than one conformeric species. The +11 ions (*m/z* 1079.2), which appear at pH 2.6 as three peaks centered on drift times of 5.1, 6.3, and 7.7 ms, have been fitted to three Gaussian distributions (Figure 4b). The +11 charge state ions begin to appear in spectra acquired at pH < 5 and are detected as three resolved peaks whose drift times remain constant during the pH titration down to pH 2.60. Although these peaks do tend to broaden slightly with decreasing pH, they are consistently symmetrical in appearance. The +13 ions (*m/z* 913.3) give rise to a predominant signal of drift time 5.3 ms, together with a much smaller distribution of ions with a drift time of ∼6.3 ms; this trace can also be described by two discrete Gaussian distributions.

Using these Gaussian distributions, which are in good agreement with the visible conformers depicted in the Driftscope plots illustrated in Figure 3, and assuming that the more compact species have a shorter drift time than the less compact species of the same charge state, the relative contribution to each conformer from every charge state under all pH conditions was calculated as a fraction of the total peak area of the spectrum from which it originated. Examples of these results are displayed for the charge state ions +8, +11, and +13 (Figure 4d, e, and f, respectively). Thus the +8 charge state ions are shown as being associated solely with Species A (filled circles) and decrease significantly in intensity below pH ∼ 4.5 (Figure 4d). The +11 ions contribute mainly to the more expanded Species B (filled squares) below pH 5 as well as, to a lesser extent, the more compact Species A (filled circles) and the least compact conformer, Species C (open squares) (Figure 4e). The +13 ions contribute to Species B (filled squares) and Species C (open squares) below pH 4 (Figure 4f).

**Figure 5:**
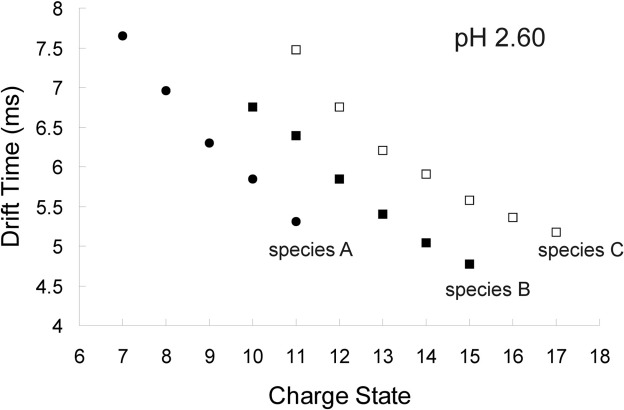
Charge state ions versus drift time graphs of the Gaussian distribution means fitted to the data acquired at pH 2.60. The native-like state (Species A) is shown as ●, the lesser folded state (Species B) as ■, and the least folded species (Species C) as □.

To assign the individual Gaussian distributions to a discrete protein conformation, the mean drift time value of each was plotted against the charge state from which it originated for the data acquired at each pH; the data acquired at pH 2.60 are illustrated in Figure 5. The mean values were largely invariant over the pH range under investigation. Three distinct linear series, referred to as Species A, B, and C, are observed despite having overlapping charge state distributions. For a given charge state, the distribution with the highest mobility has been assumed to correspond to the most folded conformeric species. From this graph it can be seen that for a given conformer, the ions with the higher charge states have shorter drift times than those with lower charge states, as would be expected in a conventional ion mobility experiment. Thus at pH 2.60, three conformeric distributions have been articulated comprising the native/native-like state (Species A, with contributions from the +7 to +11 charge state ions; filled circles), a more expanded conformer (Species B, with contributions from the +10 to +15 charge state ions; filled squares), and the least compact conformer (Species C; +11 to +17 charge state ions; open squares) (Figure 5).

**Figure 6:**
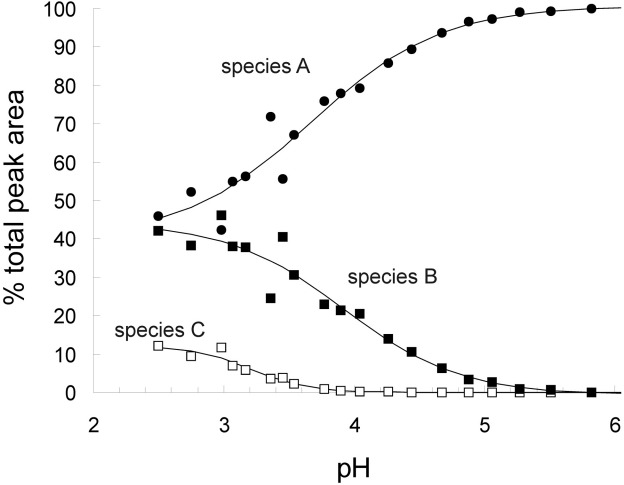
The percentage conformer-specific contribution of each charge state shows that the denatured ensembles at pH 4.5 and below are mixtures of different conformational states. The peak areas associated with each conformeric species were summed and normalized to the total area of all peaks in the data acquired at the given pH, and plotted as pH versus percentage peak area. Species A unfolds at pH < 5 (●), Species B becomes populated at pH < 5 (■), and Species C increases in intensity at pH < 3.6 (□). The curves were fitted to the data using OriginPro 7.5 (Originlab, Northampton, MA) to enhance clarity.

Thus, acid-unfolding of the protein β_2_m and the individual appearance and disappearance of the various conformational states can be monitored directly during the acid titration of the protein; Figure 6 shows the conformer-specific contribution of each charge state throughout the pH titration obtained by summation of the peak areas associated with each of the three conformeric species delineated, followed by normalization to the total peak area at each pH, thus permitting an estimation of the relative proportions of three conformeric states, Species, A, B, and C. By default, this method assumes that ions of different charge states ionize to the same efficiency. A number of distinct trends is observed in the acid titration of β_2_m. As previously reported, β_2_m is predominantly in a native state between pH 7 and 4.5 [31]: here native state (Species A) unfolding can be seen to occur below pH ∼ 4.8, whilst the more expanded conformer (Species B) is detected at pH < 5 with maximum occupancy at pH ∼ 3.0. The least compact conformation (Species C), depicted by a wide range of charge states (+10 to +17) due to its dynamic nature, is seen to increase in intensity below pH 3.6. Thus discrete conformational states of β_2_m corresponding to copopulated species consistent with folded, partially folded and acid-unfolded populations can be physically separated and identified.

However, on inspection of Figure 6, Species A appears to be present at ∼45% of the total ensemble at pH 2.5. As it has been shown from earlier studies that little natively-folded protein remains at such low pH [47], this observation appears anomalous. On closer inspection of the +8 charge state ions, it is apparent that the peak broadening observed concurrent with decreasing pH shows a dramatic increase at pH ∼ 4.0 (Figure 4a inset). A similar trend is observed for the +7 charge state ions (data not shown). It is at this pH that the protein begins to undergo significant unfolding [33, 47]. A plausible explanation for this is that the +7 and +8 charge state ions are associated only with the native conformer (as in Figure 3a) whilst the +9, +10, and +11 charge state ions, which appear at lower pH “in-line” with the native conformer (Figure 3b, c, and d), define a second conformer with very little difference in cross sectional area to the native protein but with a higher propensity for protonation. This explanation is supported by the fact that if the +7 and +8 species alone are considered representative of the folded protein conformer, then at pH 2.5 their contribution to the total ensemble of species is ∼15%, which is a far more realistic value based on previous findings [33, 47]. Thus, this indicates that four conformeric families have been detected, two within the group of ions labeled Species A, one of which corresponds to the native protein and the other to a more extended species, together with Species B and Species C, which are consistent with further protein unfolding, although higher resolution of the ESI-IMS-MS data is required to define the Species A ensemble better.

#### Comparison of the conformational properties of wild-type β_2_-microglobulin with protein variants

**Figure 7:**
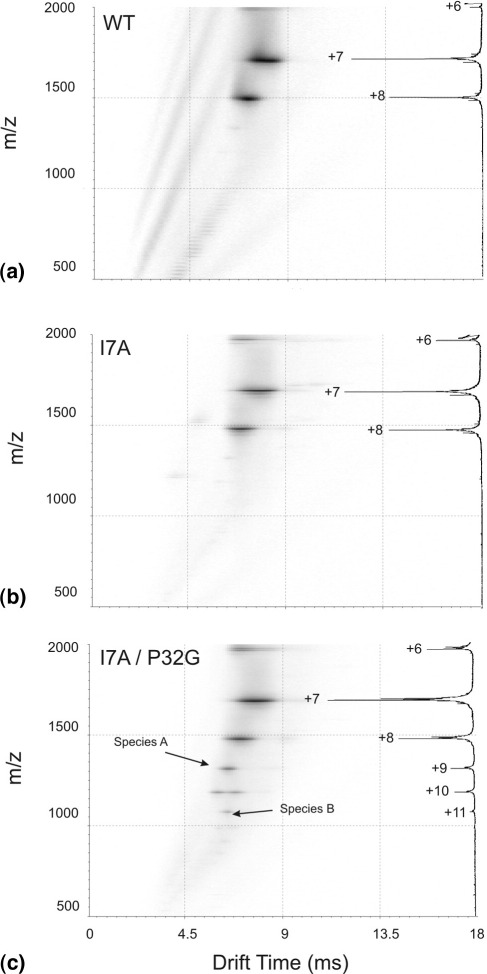
(**a**)–(**c**): ESI-IMS-MS Driftscope plots showing drift time (x axis) versus *m/z* (y axis) for β_2_m and variants at pH 6.8. (**a**) wild-type β_2_m, (**b**) single mutant I7A, and (**c**) double mutant I7A/P32G. Insets at the right hand side: the summed, full scan *m/z* spectra of each protein showing the charge state ions detected.

To gauge the effectiveness of ESI-IMS-MS to compare similar protein sequences of varying stability, a number of well-characterized β_2_m mutants that are destabilized to varying extents compared with the wild-type protein were selected specifically due to their folding characteristics, stabilities, and propensities for forming amyloid fibrils and examined under identical ESI-IMS-MS conditions at pH 6.80. Two of these are highlighted here: the single mutant I7A and the double mutant I7A/P32G (Figure 2). For the purposes of this investigation, only the monomeric forms of the proteins have been studied, and any high *m/z* oligomeric species observed were not included in this comparison. I7A is destabilized significantly relative to wild-type β_2_m, as shown by equilibrium denaturation studies [33], but is not able to form amyloid fibrils at neutral pH in significant yield without the addition of seeds [28, 49]. Despite its significant destabilization, the Driftscope data for the I7A mutant (Figure 7b) were very similar to those of wild-type β_2_m (Figure 7a), consistent with the view that this protein does not populate partially folded states at this pH, and indicating the population of one major conformer delineated by +7 and +8 charge state ions. The drift times of these charge state ions were in accord with the drift times of the corresponding ions detected for the native, wild-type protein (Figure 7a). These data fit well with the supposition that the I7A variant retains a native-like structure at neutral pH, despite its destabilization relative to wild-type β_2_m [33].

By contrast, the double mutant of β_2_m containing both I7A and P32G mutations, which has recently been shown to form de novo amyloid fibrils rapidly at high yields under neutral conditions in the absence of any cofactors (Jahn, T. J. and Radford, S. E., unpublished data), gave strikingly different results. The Driftscope plot of this variant indicates that folded and more extended conformations, consistent with Species A and B observed for wild-type β_2_m (Figure 3), are both populated at pH 6.8 (Figure 7c) and these data are very similar to the data obtained for the denatured wild-type protein at pH 4.28 (Figure 3b). These results support the supposition that I7A/P32G populates partially folded conformational states under native conditions, a feature which is linked to de novo fibril formation, and illustrate that the existence of these particular species can be quickly confirmed and characterized by ESI-IMS-MS.

These data thus highlight the power of ESI-IMS-MS to reveal, resolve, and quantify copopulated species in a single experiment that would otherwise be difficult to discern using other biophysical methods and indicate the limits of resolution when distinguishing between minor changes in protein conformational states. An additional feature of this method is the low sample consumption (ca. 1 μg) required to generate these data, i.e., far less than that required by other biochemical and biophysical techniques such as NMR, CD, and equilibrium denaturation studies, which all require >500 μg.

## Conclusion

The study of ESI-MS charge state distributions and their relationship to protein conformeric forms is well documented in the literature [9, 11, 22]. The use of deconvolution methods to resolve these distributions further with the aim of quantifying the conformer populations within these charge state distributions has also been reported [13, 14]. Here, by use of a novel biophysical technique and well studied model systems, we have demonstrated that ESI-IMS-MS can be used to directly monitor and separate conformeric forms within an ensemble of species of the protein standard cytochrome *c* and the amyloidogenic protein β_2_m based on their cross sectional area. These results indicate ESI-IMS-MS to be a potentially powerful tool capable of rapidly revealing rare conformations of proteins populated under different solution conditions and of characterizing these species based on their charge state ions and cross sectional areas.

The ESI-IMS-MS results presented here demonstrate that β_2_m populates a number of distinct conformational families encompassing the native, one or more partially folded, and the more acid-unfolded states, and that the relative populations of these copopulated species can be monitored individually and quantified as a function of pH. An interesting feature arising from these analyses is whether the higher charge state ions associated with Species A, which appear on protein acidification, are a result of more extensive protonation of the folded conformer or indicate an additional conformer of very similar cross sectional area. The latter postulation is strengthened by the fact that the +7 and +8 charge state ions associated with the folded conformer show significant peak broadening accompanied by an asymmetric appearance under more acidic conditions, but further investigation and higher data resolution are required to confirm beyond doubt that a second, partially folded conformer has been observed. We have also shown that substantial global destabilization of the native fold of wild-type β_2_m by a single amino acid mutation of I7A does not alter significantly its cross sectional area or result in the significant population of partially folded or acid-unfolded conformations at neutral pH. In contrast, the double mutation I7A/P32G results in the population of coexisting partially folded species at pH 6.8, under which conditions the protein is capable of de novo fibril formation. These partially folded species are more akin to the ESI-IMS-MS data observed for wild-type β_2_m at pH 4.28, and as such may well be linked to the known enhanced amyloidogenicity of this double mutant.

Coupled with real time assays of amyloid formation, the ability of ESI-IMS-MS to resolve different conformational states of monomeric and high oligomeric forms promises to make this technique central to the analysis of protein aggregation and other heterogeneous assembly reactions.
